# Developing key performance indicators for guaranteeing right to health and access to medical service for persons with disabilities in Korea: Using a modified Delphi

**DOI:** 10.1371/journal.pone.0208651

**Published:** 2018-12-07

**Authors:** Bomyee Lee, So-Youn Park

**Affiliations:** Department of Medical Education and Medical Humanities, Kyung Hee University School of Medicine, Dongdaemun-gu, Seoul, Korea; Medical University Graz, AUSTRIA

## Abstract

Recently, the Act on Guarantee of Right to Health and Access to Medical Service for Persons with Disabilities was implemented to legally define the health care system for persons with disabilities (PWDs) and the guarantee of access to medical care in Korea. This study aimed to develop specific goals and performance indicators to establish a system to guarantee right to health and access to medical service for PWDs. The first procedure was the establishment of the performance indicators, and the second was the content validity verification of the established performance indicators. To establish the performance indicators, we used the policy indicators of the government to improve the health of the Korean people. The indicators that needed to be newly developed were established based on literature review and expert consultation. Three Delphi surveys were conducted to verify the content validity of the established performance indicators. The content validity index (CVI) was obtained for the importance and possibility of the performance indicators. The indicators using the existing policy indicators are “proportion of public health centers” and “rate of health checkup of PWDs,” and newly developed indicators are “establishment of facilities for PWDs in health care facilities (buildings and personnel)” and “diagnosis of autism spectrum disorder in early childhood (average age and awareness).” The final performance indicators consist of a total of six areas, 22 sub-areas, and 40 individual indicators. The final performance indicators in this study can be used as basic data for continuously identifying the health status of PWDs in Korea and establishing the national policy for their health promotion. This study is also expected to serve as a framework to guarantee the right to health and access to medical service for PWDs rather than simply containing declarative content.

## Introduction

According to 2011 World Health Organization (WHO) data, over a billion people (or about 15% of the world’s population) are estimated to be living with disability [1]. In Korea, the number of persons with disabilities (PWDs) registered nationwide is about 2.54 million people (or about 4.9% of the total population) [2]. The PWD population is expected to increase steadily owing to population aging, increased incidence of chronic diseases, various accidents, and disasters [3].

PWDs are more susceptible to diseases, and the incidence of chronic diseases in this group is high. Specifically, about 77% of Korean PWDs suffer from such chronic diseases as hypertension and diabetes, and their mortality rate is more than four times that of the total population [3]. However, health care services and support systems for PWDs in Korea remain insufficient, and the health disparity between people with and without disabilities persists because of insufficient health care management system [4,5].

Since the 1980s, the international community has begun recognizing the health disparity issue, acknowledging that health problems are not the responsibility of individuals and families but should be solved by society [6]. In line with these international trends, Korea enacted related laws, such as the Act on Welfare of Persons with Disabilities and Act on the Prohibition of Discrimination against Persons with Disabilities, Remedy against Infringement of their Rights. However, these laws only prescribe content related to the welfare promotion of PWDs and their participation in social activities, but not health and health care services [7]. In December 2015, the Act on Guarantee of Right to Health and Access to Medical Service for Persons with Disabilities (Act on Right to Health for Persons with Disabilities) was enacted to define legally the health care system for PWDs and the guarantee of access to medical care; the Act was implemented in December 2017.

According to Article 6, the main content of the Act on Right to Health for Persons with Disabilities, the Minister of Health and Welfare has to establish Comprehensive Plans for Health Care for Persons with Disabilities (Comprehensive Plan) every five years. Specifically, a comprehensive plan shall include the objectives and direction-setting for health care programs, a plan and methods for implementing health care programs, training of specialists, education, and health management according to gender characteristics [8]. However, the requirements are specified only at the item level, and the details of each are not comprehensive and systematic. For this Act to be an act for the actual promotion of the health of PWDs, rather than declarative content, specific goals and performance indicators for each detail must be presented in a comprehensive and systematic manner.

This study aimed to develop specific goals and performance indicators for each detail of the Comprehensive Plan, and then use them as base data to establish health care management policy for PWDs.

## Methods

### Sources to construct the indicators

#### Using existing policy indicators

To construct the performance indicator, we analyzed the representative health policies currently implemented in Korea to improve people’s health and the performance indicator developed to evaluate the performance of the policy. Specifically, we analyzed the Comprehensive Plan for National Health Promotion (Health Plan 2020), Comprehensive Policy Plan for Persons with Disabilities, and others. Among them, applicability and available indicators were selected as the performance indicators of this study.

**Fig 1:**
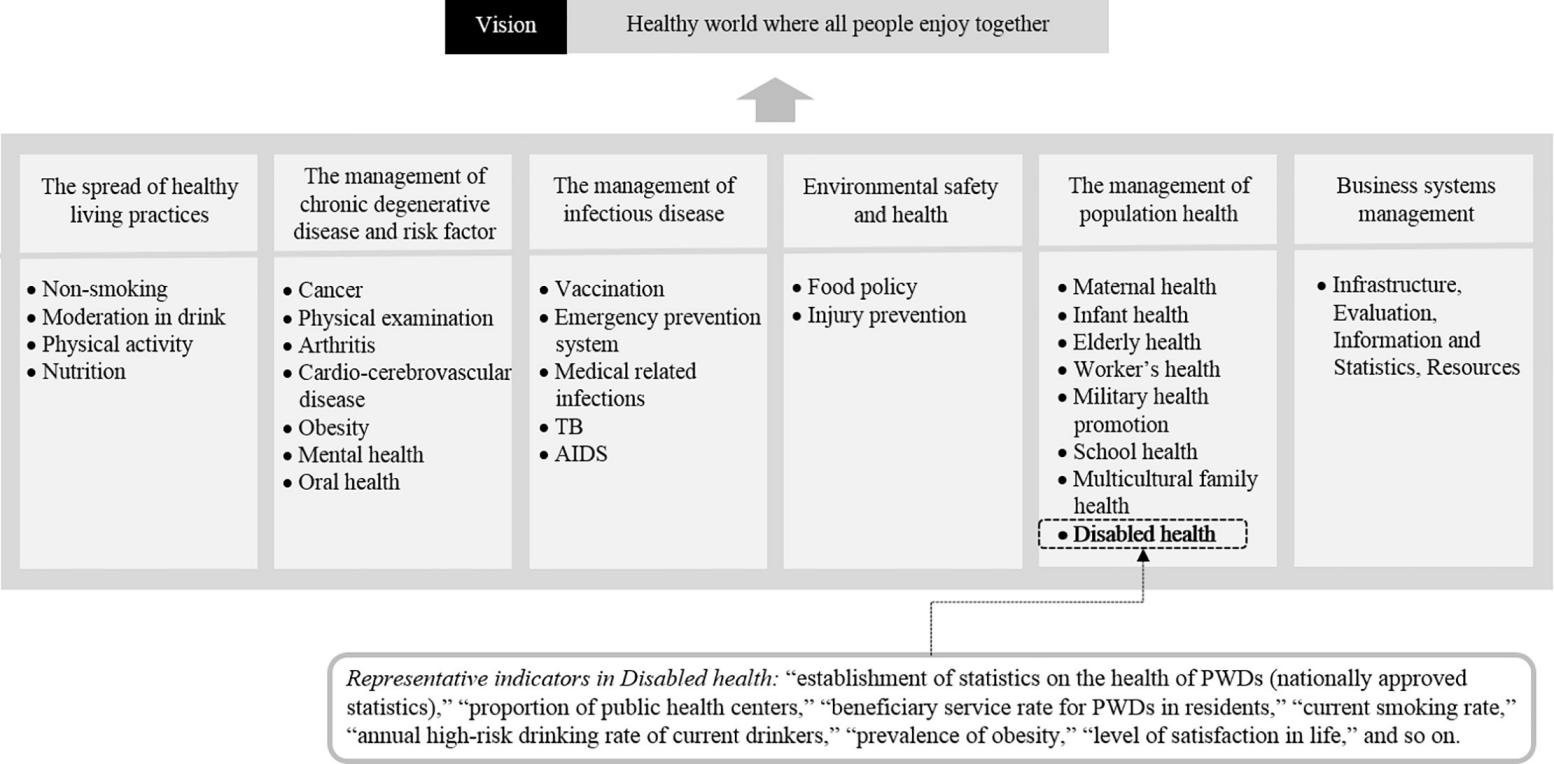
Comprehensive plan for National Health Promotion (Health Plan 2020).

Health Plan 2020 is a comprehensive government mid- to long-term comprehensive plan that introduces the direction of the health promotion policy for health promotion and disease prevention of Korean people every five years. The Health Plan 2020 is composed of six fields, 27 tasks, and 367 performance indicators to achieve the goal of extending the life span of the Korean people and improving health equity [9]. Article 6 of Act on Right to Health for Persons with Disabilities requires that the content of the Health Plan 2020 must be included when establishing and implementing a Comprehensive Plan [8]. In other words, the Comprehensive Plan should be established in the same direction as the goal of the Health Plan 2020 because the latter is a key plan that is the backbone of Korean health policy. Therefore, among the performance indicators pursued by the Health Plan 2020, the indicators applicable to this study were selected as the performance indicators (Fig 1). The performance indicators of this study constructed using the performance indicators of the Health Plan 2020 are as follows: (1) establishment of statistics on the health of PWDs (nationally approved statistics), (2) proportion of public health centers, (3) beneficiary service rate for PWDs in residents, (4) current smoking rate, (5) smoking cessation attempts by current smokers, (6) annual high-risk drinking rate of current drinkers, (7) a month’s drinking rate, (8) physical activity practice rate, (9) regular meal rate, (10) rate of health checkup of PWDs, (11) prevalence of obesity, and (12) level of satisfaction in life.

The Comprehensive Policy Plan for Persons with Disabilities is a plan designed to promote the interests and welfare of PWDs, mainly designed to support welfare services rather than plans for the health promotion of PWDs [10]. Among the performance indicators promoted by the 4^th^ Comprehensive Policy Plan for Persons with Disabilities, this study used constructed “establishment of statistics on the health of PWDs (nationally approved statistics)” and “physical activity practice rate.”

#### Newly developed performance indicators

The use of existing health policy performance indicators alone cannot provide key areas, specific goals, or specific performance indicators for each of the details of the Comprehensive Plan. To construct indicators that need to be newly developed, we first analyzed the details that should be included in the Comprehensive Plan. Article 6 of the Act on Right to Health for Persons with Disabilities should include information on the education and training of specialists required for the management of PWDs, gender characteristics, and management of health care for women with disabilities, such as maternity protection [8]. Therefore, this study constituted performance indicators related to training and education specialists, women with disabilities, and pediatric developmental disabilities.

The performance indicators of this study, constructed according to Article 6 of the Act on Right to Health for Persons with Disabilities, are as follows: (1) training and improvement of awareness of specialists in PWDs (regular curriculum, special curriculum, and medical personnel), (2) implementation of tracking inspection support, (3) financial support for early detection of developmental disabilities, (4) diagnosis of autism spectrum disorder in early childhood (average age and awareness), (5) regular screening rate during pregnancy, (6) infant mortality rate, (7) maternal mortality rate, (8) rate of breast cancer screening, (9) rate of cervical cancer screening, and (10) sexual education experience.

Through review of the literature, we analyzed the problems and risk factors in the field of health care currently faced by PWDs in Korea and the indicators that need to be further developed. PWDs in Korea have a higher prevalence of chronic diseases, such as hypertension, diabetes, dental caries, and periodontal diseases. In addition, depression and stress levels are high, and the rate of suicidal ideation is also exceedingly high owing to a complication of economic difficulties and pessimism [11–13]. Currently, national research data, such as the National Survey of the Disabled Persons and Korea National Health and Nutrition Examination Survey, identify the health status of PWDs in Korea. However, the National Survey of the Disabled Persons, conducted every three years, is not an actual measurement but a self-reporting survey. In addition, PWDs living in facilities are excluded from the survey. Indeed, various limitations that prevent the understanding of the actual health level of PWDs in Korea have been pointed out [11]. In the Korea National Health and Nutrition Examination Survey, a question on disability registration was added to the survey questionnaire for the sixth period (2013–2015), thereby making it possible to analyze the data according to disability status. However, the questionnaires were originally intended for the entire population, and not designed to assess the level of health of PWDs, setting a limit to understanding of the characteristics of disabilities [14]. Therefore, this study aimed to establish systematically the health-related data of PWDs to shed light on the level of health of PWDs by creating indicators that can reveal their level of chronic disease. The performance indicators developed are as follows: (1) prevalence of accidents/poisoning, (2) rate of cancer screening of PWDs, (3) prevalence of hypertension, (4) prevalence of diabetes, (5) prevalence of dental caries, (6) prevalence of periodontal disease, (7) level of oral hygiene, (8) level of oral care, (9) depression level, (10) stress level, and (11) level of suicide attempts.

Finally, opinions on the performance indicator items were collected through three expert advisory meetings and a representative meeting of six PWD groups. In this process, it was suggested that a medical facility that is accessible to PWDs is needed, as well as a system that provides integrated information on the health of PWDs. In response to these opinions, this research team newly added indicators for the following: (1) establishment of statistics on the health of PWDs (statistics on characteristics of disabilities), (2) establishment of facilities for PWDs in health care facilities (building and personnel), and (3) strengthening provision of health information services. The initial performance indicators were then constructed. The initial performance indicators are composed of six areas, 21 sub-areas, and 40 individual indicators (S1 Table).

### Participants of the modified Delphi

Three Delphi surveys were conducted to confirm the content validity of the initial performance indicators. The Delphi survey is a research method that draws consensus through gathering opinions of experts with expertise and knowledge on research subjects when decision-making based on objective, accurate information is difficult [15]. In addition, the results of the Delphi survey are entirely influenced by expert knowledge, opinions, and intuition, and as such, the selection of expert panels is critical [16]. The panel of experts in this research consisted of associate professors or higher in their field of study and team leaders or higher in related organizations, selected with respect to the purpose and characteristics of this study. Specifically, the panel consisted of 26 academics, from such fields as rehabilitation medicine, social welfare, dentistry, and preventive medicine, and 3 experts working as practitioners in the field of PWD study. More than 65% of them had more than 10 years of experience in their respective field of study. After the recruitment letter was sent to the selected panel of experts, those who agreed to participate in the research were sent the letter of consent and Delphi survey through email. Of the 31 who agreed to participate in the survey, 29 (93.5%) responded to the first, second, and third surveys. This study’s procedures, including the consent process, were approved by the Kyung Hee University Institutional Review Board, Korea (Approval No. KHSIRB-17-034RA).

### Procedures of the modified Delphi

A total of three Delphi surveys were conducted through email from May to October 2017 for selected panelists. The first Delphi survey was based on the initial performance indicators; the importance and possibility of each indicator was evaluated by a four-point Likert scale (1 point: not at all important / no possibility, 2 points: not important / low possibility, 3 points: important / high possibility, 4 points: very important / very high possibility). “Importance” referred to the level of significance of the indicators in monitoring the health status of PWDs and suggesting a strategic direction, whereas “possibility,” whether the indicators could accurately quantify the measured and collected data.

The second Delphi survey was conducted based on the results of the first Delphi survey to inform the panel of the indicators adopted, revised/supplemented, and deleted for their reference in reevaluation. The participants were asked to describe freely their opinions on each indicator. As with the first Delphi survey, the “importance” and “possibility” of each indicator was evaluated on a 4-point Likert scale.

The third Delphi survey also provided guidance on the results of the second Delphi survey and asked for individual opinions. Likewise, as in the first and second Delphi surveys, the “importance” and “possibility” of each indicator was evaluated on a 4-point Likert scale.

**Fig 2:**
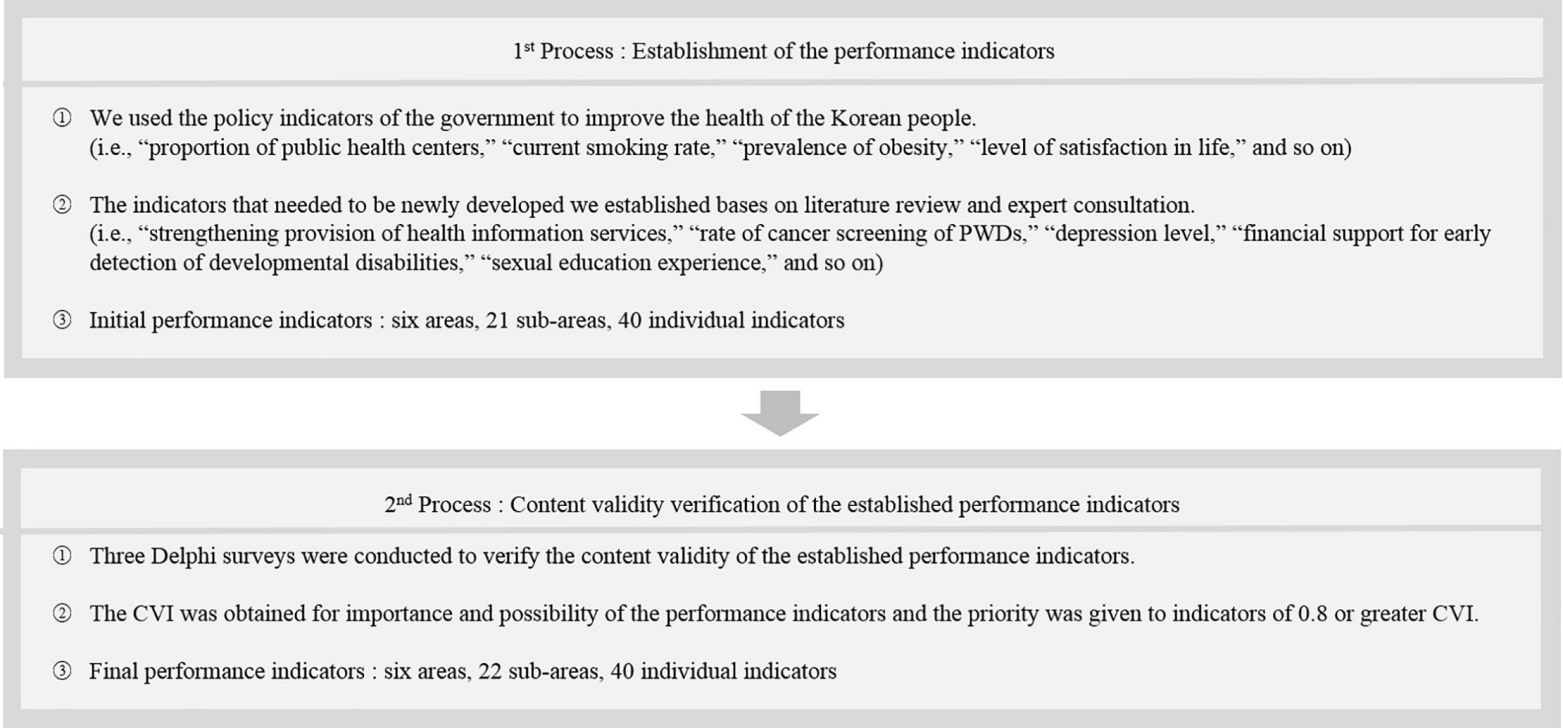
Establishment of the performance indicators and modified Delphi process.

The content validity index (CVI) was calculated for each survey stage to verify the content validity of each indicator. According to the criteria suggested by Lynn [17], indicators with a CVI of 0.8 or more were adopted, those with 0.5 to less than 0.8 were discussed (mediated differences of opinions), and those with less than 0.5 were dropped. The final selected indicators were not repeatedly measured (e.g., “the rate of health checkup of PWDs” was selected in the first Delphi survey, so it was not re-measured in the second Delphi survey). However, for the unadjusted indicators, the focus was on continuing the next Delphi round and drawing consensus from the expert panels. In principle, indicators were selected only if the CVI value was 0.8 or greater in both importance and possibility of the indicator. However, a number of indicators were selected as the final performance indicators even if the CVI for the possibility was less than 0.8 (Fig 2).

## Results

### First Delphi survey results

The indicators with CVI of 0.8 or more in terms of importance and possibility are as follows: “training and improvement of awareness of specialists in PWDs (regular curriculum, special curriculum, and medical personnel),” “rate of health checkup of PWDs,” “rate of cancer screening of PWDs,” “financial support for early detection of developmental disabilities,” “regular screening rate during pregnancy,” “rate of breast cancer screening,” and “rate of cervical cancer screening.”

The indicator for “level of oral care” was excluded because the CVI for possibility was less than 0.5. In addition, indicators with high importance (CVI ≥ 0.8) but low possibility (0.5 ≤ CVI < 0.8) were added with the measurement basis and additional explanation of the statistical data that can be utilized for the second Delphi survey by the panel. The indicators “rate of dental examinations,” “sealant utilization rate,” and “scaling utilization rate” were newly added, reflecting the opinions of the expert panel.

### Second Delphi survey results

The indicators with CVI of 0.8 or more in terms of importance and possibility are as follows: “beneficiary service rate for PWDs in residents,” “establishment of facilities for PWDs in health care facilities (buildings and personnel),” “strengthening provision of health information services,” “prevalence of obesity,” “prevalence of hypertension,” “prevalence of diabetes,” “rate of dental examination,” “infant mortality rate,” and “maternal mortality rate.”

“Sealant utilization rate” and “scaling utilization rate” had low CVI scores in importance and possibility, and were thus excluded. In addition, we added the indicators “prevalence of osteoporosis” and “rate of medical examination in children with disabilities,” reflecting the opinion of the expert panel (S2 Table). Specifically, they shared the following major opinions.

“I wish there were more indicators for the diagnosis rate or incidence of osteoporosis. Women with disabilities are suffering from musculoskeletal disorder at an earlier age than persons without disabilities.” (Social welfare expert).

“In Korea, students with disabilities are legally required to undergo school medical examinations like students without disabilities, but medical institutions have avoided medical examinations for students with disabilities for a variety of reasons. As a result, medical examinations for students with disabilities are in blind spots.” (Preventive medicine expert).

### Third Delphi survey results

The following indicators scored a CVI of 0.8 or more on importance and possibility: “establishment of statistics on the health of PWDs (nationally approved statistics and statistics on characteristics of disabilities),” “proportion of public health centers,” “current smoking rate,” “annual high-risk drinking rate of current drinkers,” “physical activity practice rate,” “number of meals per day,” “prevalence of accidents/poisoning,” “prevalence of dental caries,” “prevalence periodontal diseases,” “level of oral hygiene,” “level of satisfaction in life,” “rate of follow-up examinations,” “rate of medical examination in children with disabilities,” and “prevalence of osteoporosis.”

The “smoking cessation attempts by current smokers” and “a month’s drinking rate” showed CVI values of 0.8 or greater for possibility but lower values for importance. They were thus removed from the final performance indicators in this study. Although the CVI for possibility was lower than 0.8 but higher for importance (CVI ≥ 0.8) for “depression level,” “stress level,” “level of suicide attempts,” “diagnosis of autism spectrum disorder in early childhood (average age and awareness),” and “sexual education experience,” these were included in the final performance indicator.

### Selection of final performance indicators

This study conducted three Delphi surveys based on initial performance indicators and then selected the key areas and final performance indicators based on the collected results. The final performance indicators consist of a total of 6 areas, 22 sub-areas, and 40 individual indicators; the details are shown in Table 1.

**Table 1:** Final performance indicators.

Area	Sub-area	Indicator	Definition of indicator
**Health care management**	Basis for improving the level of health of PWDs	Establishment of statistics on the health of PWDs (nationally approved statistics)	Expansion of health statistics for PWDs among nationally approved statistics
		Establishment of statistics on the health of PWDs (statistics on characteristics of disabilities)	Establishment of health statistics reflecting types of disability
	Medical accessibility for PWDs	Proportion of public health centers	Percentage of public health centers that conduct community-based rehabilitation projects among public health centers nationwide
		Beneficiary service rate for PWDs in residents	Percentage of PWDs receiving community-based rehabilitation services from residences
		Establishment of facilities for PWDs in health care facilities (buildings)	Information desk for PWDs and common checklists
		Establishment of facilities for PWDs in health care facilities (personnel)	Satisfaction of PWDs’ accompanying services using volunteer workforce of health care institutions
	Accessibility to health-related information for PWDs	Strengthening provision of health information services	Development and provision of standardized health information contents for PWDs
	Training and education specialists	Training and improvement of awareness of specialists in PWDs (regular curriculum)	Curriculum for understanding and education of disabilities in the process of specialized training for medical personnel and others
		Training and improvement of awareness in specialists in PWDs (special curriculum)	Mandatory education in treating PWDs in the process of specialized training for medical personnel and others
		Training and improvement of awareness in specialists in PWDs (medical personnel)	Obligation to offer programs on disability education for medical personnel when certifying medical institutions
**Practice of healthy lifestyle**	Non-smoking	Current smoking rate	Rate of PWDs aged 12 and older who currently smoke cigarettes daily or occasionally
	Moderation in drink	Annual high-risk drinking rate of current drinkers	Rate of PWDs aged 12 and older who have had alcohol during the last year and had over 7 glasses per occasion for men (5 for women), or who drink more than twice a week
	Physical activity	Physical activity practice rate	Rate of PWDs who practice physical exercise (those who exercise more than two to three times a week for 30 minutes or more per week for activities other than rehabilitation)
	Nutrition	Number of meals per day	Number of meals per day for PWDs
	Accidents/poisoning	Prevalence of accidents/poisoning	Incidence of accidents or poisoning that had to be treated at hospitals or emergency rooms for the past one year after disability onset
	Health checkup	Rate of health checkup of PWDs	Whether a health checkup has been conducted during the last two years
		Rate of cancer screening of PWDs	Whether a cancer screening has been conducted during the last two years
**Management of chronic diseases**	Obesity	Prevalence of obesity	Prevalence of obesity in PWDs aged 20 and older
	Hypertension	Prevalence of hypertension	Percentage of PWDs diagnosed with hypertension
	Diabetes	Prevalence of diabetes	Percentage of PWDs diagnosed with diabetes
	Oral health	Prevalence of dental caries	Decayed-Missing-Filled-Teeth index
		Prevalence of periodontal disease	Community Periodontal Index of Treatment Needs
		Level of oral hygiene	Patient Hygiene Performance index
		Rate of dental examination	Whether an oral check-up has been conducted during the last 2 years
	Mental health	Depression level	Whether one experienced sadness or despair enough to interfere with daily life for more than two consecutive weeks for the past one year
		Stress level	Level of stress experienced in daily life
		Level of suicide attempts	Whether one has attempted suicide in the last year
**Quality of life**	Life satisfaction	Level of satisfaction in life	Percentage of PWDs who are “very satisfied” or “satisfied” with life
**Children with disabilities**	Pediatric development	Rate of follow-up examinations	The rate of follow-up examinations conducted on infant and toddlers who were asked to return for a “follow-up”
		Financial support for early detection of developmental disabilities	Financial support for the early detection of developmental disabilities in infants and young children who were asked to receive “in-depth examination”
	Diagnosis of autism spectrum disorder in early childhood	Diagnosis of autism spectrum disorder in early childhood (average age)	Average age of diagnosis of the neuropsychiatric code in Korean standard disease sign classification
		Diagnosis of autism spectrum disorder in early childhood (awareness)	Enhancing public awareness of the importance of early diagnosis and improved awareness of autism spectrum disorder in children
	Medical examination	Rate of medical examination	Whether a medical examination has been conducted on students with disabilities attending specialized schools
**Women with disabilities**	Health	Regular screening rate during pregnancy	Percentage of women with disabilities who received at least one regular screening after pregnancy was confirmed
		Infant mortality rate	Number of deaths (within one year after birth) divided by number of births in the year shown per 1,000 births
		Maternal mortality rate	Number of maternal deaths per 100,000 births
		Prevalence of osteoporosis	Prevalence of osteoporosis in women with disabilities over 40 years of age
	Cancer screening	Rate of breast cancer screening	Whether a breast cancer screening has been conducted on women with disabilities over the age of 40 during the last two years
		Rate of cervical cancer screening	Whether a cervical cancer screening has been conducted on women with disabilities over the age of 40 during the last two years
	Sex education	Sexual education experience	Percentage of women with disabilities who have received sex education (pregnancy, giving birth, birth-control, etc.)

## Discussion

Korean laws for PWDs have mainly focused on the promotion of PWDs’ welfare and participation in social activities. The Act on Right to Health for Persons with Disabilities is of great significance in that the rights to health of PWDs are stated in the law for the first time and their healthcare access is legally guaranteed. For factors hindering the health care access of PWDs, previous studies have identified the following: economic hardship, physical difficulties (i.e., difficult to visit a health care facility owing to transportation problems), difficulty in accessing information, medical staff’s negative perception of PWDs, among others [18–20]. According to a survey in Korea, the most uncomfortable experience by PWDs in using hospitals/clinics and receiving medical care is “physicians’ lack of understanding of disability and insufficient consideration” (34.8%), followed by “lack of PWD-friendly facilities in hospitals/clinics” (26.8%) and “difficulties in communication and information access” (14.1%) [21]. In the present study, such indicators as “establishment of facilities for PWDs in healthcare facilities (buildings and personnel),” “strengthening provision of health information services,” and “training and improvement of awareness of specialists in PWDs (regular curriculum, special curriculum, and medical personnel)” were developed to increase the health care accessibility of PWDs. Indeed, many policy studies conducted so far in Korea have largely been insufficient in terms of reflecting health assessment factors pertinent to PWDs. In this context, the present study is of significance in that to increase the health care accessibility of PWDs by considering various realistic difficulties faced by PWDs and their characteristics, new performance indicators were developed and evaluated for representativeness and validity.

Currently, various movements are being made gradually at the government level to guarantee health care accessibility of PWDs in Korea. Hospitals/clinics are required to set up an information desk devoted solely for PWDs, and government agencies are required to operate health information sites for PWDs to provide them with quality health information [8]. Furthermore, to develop personnel specialized in PWDs and improve public perception of PWDs, the Ministry of Health and Welfare recently announced to 11 health care-related associations, including the Korean Medical Association, the availability of “training for healthcare professionals on the health rights of PWDs” beginning in 2018 [22]. To establish and execute policies and projects such as these, evidence-based performance indicators that are objective and can be used for continuous monitoring should be developed. From this perspective, the performance indicators proposed in this study will be helpful in suggesting the direction for various national policy projects currently planned to improve the health care accessibility of PWDs.

In Korea, women with disabilities face problems as women and as PWDs, as well as problems with complex characteristics arising from the combination of the two. The health issues of women with disabilities have not received appropriate concern in Korea, taken as too sensitive to handle [23]. In conducting the present study, too, indicators in the area of women with disabilities underwent a number of revisions and modifications. In the beginning, topics that could include major issues like “abortion rate,” “safe contraceptive use rate,” and “sexual violence rate” were selected as initial indicators, but they were excluded during the expert advisory meetings and group meetings with PWDs. The reasons for excluding these indicators were “sensitive to handle” and “difficult to measure in reality.” Eventually, indicators in the area of women with disabilities came to consist of “regular screening rate during pregnancy,” “infant mortality rate,” “maternal mortality rate,” among others, after referring to the WHO data on reproductive health indicators [24]. To ensure continuous monitoring based on these indicators, an integrated database that combines raw data spread across various organizations should be constructed. However, at present, the statistics on the PWD population cannot be established for still being rudimentary. The construction of the aforementioned database requires a system linking the raw data sources spread across the Ministry of Health and Welfare, Korean National Health Insurance Service, Statistics Korea, and Korean National Rehabilitation Center, which can operate and manage them in an integrated fashion. The integrated data could be used to establish the foundation for improving the health level of PWDs by identifying the status of registered PWDs and computing health statistics by disability characteristic. With the integrated data, it would be possible to compute annual health statistics for PWDs, which will in turn enable continuous monitoring of the health status of PWDs.

There are a few principles to consider in developing performance indicators. The Public Health Agency of Canada proposed such principles as “data are collected regularly and trends can be compared over time (ongoing),” “data are available and of sufficient quality to report on or data collection can be put into place at a relatively low cost (feasible),” and “provides information that is considered to be meaningful and relevant to the target user (relevant)” [25]. Similarly, NHS Institute for Innovation and Improvement proposed principles of “importance and relevance,” “measurement possibility,” and “meaning” [26]. In the present study, these developmental principles were referenced, and importance and measurement possibility were examined for each of the indicators. That is, “whether the indicator is important in examining the health status of PWDs and suggesting the policy direction (importance)” and “whether the issue is actually possible to measure (possibility)” were simultaneously tested. After three rounds of Delphi survey, some indicators were high in CVI (0.8 or higher) in both importance and possibility. However, a few indicators had high CVIs for importance but somewhat low CVIs for possibility. These indicators require special attention to improve the problem of being difficult to measure. For example, for an indicator (i.e., sexual education experience) that can be collected through a survey, one might wonder which group of samples to choose. In the case of indicators for “depression level,” “stress level,” and “level of suicide attempts,” it is necessary select an assessment tool that can objectively and effectively measure mental health conditions. For example, using structured assessment tools such as “PHQ-9” and “Beck depression inventory” will allow for a more objective assessment and comparison of participants’ mental health to that of non-disabled people. In the case of indicators for “diagnosis of autism spectrum disorder in early childhood (average age and awareness),” institutions and funding to properly manage personal information are required. Moreover, national promotion and awareness improvement strategies are needed to improve awareness of children with autism. In the future, additional in-depth expert discussion should be held in regard to measurement tools for this indicator and the relevant survey data sources.

The present study set up key areas to be addressed in the Comprehensive Plan, and utilized and modified existing policy indicators to create performance indicators to monitor and evaluate them. New indicators were developed, necessary in the future to guarantee the health rights of PWDs and health care accessibility. However, a measurement study was not conducted on the newly developed indicators, and thus, specific suggestions were not made in the study as to the computational system and approach to utilize them. For the performance indicators developed in the present study to be applied in practice, follow-up research should be performed to test the validity more systematically and to determine specific uses of the indicators. Currently, in Korea, hospitals equipped with assistant personnel, facilities, and equipment are designated as disability-friendly health care institutions so that anyone, regardless of whether they have disabilities, can receive health checkups safely and comfortably. In addition, research is underway to develop customized medical examination items for the disabled in order to develop examination items according to disease prevention and disability characteristics. The proposed indicators for health management of PWDs can be used as basic data for establishing national policies for the disabled in Korea. In addition, it is also expected that these will enable continued understanding of the health level of PWDs and contribute to the production of statistics to improve health standards.

## Supporting information

S1 Table Initial performance indicators. (PDF)

S2 Table First, second, and third Delphi survey results. (PDF)
