# Supplementary material for: Developing key performance indicators for guaranteeing right to health and access to medical service for persons with disabilities in Korea: Using a modified Delphi
Source: PLoS One. 2018 Dec 7;13(12):e0208651. doi: 10.1371/journal.pone.0208651 (PMC6286001; doi:10.1371/journal.pone.0208651)
Supplement: S1 Table — (PDF) [file pone.0208651.s001.pdf]

S1 Table. Initial performance indicators.

Area	Sub-area	Indicator	Definition of indicator	Source to construct the indicators	
				Existing policy indicators	Newly developed performance indicators
Health care management	Basis for improving the level of health of PWDs	Establishment of statistics on the health of PWDs (nationally approved statistics)	Expansion of health statistics for PWDs among nationally approved statistics	Health Plan 2020 and 4th Comprehensive Plan for PWDs	
		Establishment of statistics on the health of PWDs (statistics on characteristics of disabilities)	Establishment of health statistics reflecting types of disability		Expert advisory meetings and a representative meeting of six PWD groups
	Medical accessibility for PWDs	Proportion of public health centers	Percentage of public health centers that conduct community-based rehabilitation projects among public health centers nationwide	Health Plan 2020	
		Beneficiary service rate for PWDs in residents	Percentage of PWDs receiving community-based rehabilitation services from residences	Health Plan 2020	
		Establishment of facilities for PWDs in health care facilities (buildings)	Information desk for PWDs and common check lists		Expert advisory meetings and a representative meeting of six PWD groups
		Establishment of facilities for PWDs in health care facilities (personnel)	Satisfaction of PWDs' accompanying services using volunteer workforce of health care institutions		Expert advisory meetings and a representative meeting of six PWD groups
	Accessibility to health related information for PWDs	Strengthening provision of health information services	Development and provision of standardized health information contents for PWDs		Expert advisory meetings and a representative meeting of six PWD groups
	Training and education specialists	Training and improvement of awareness of specialists in PWDs (regular curriculum)	Curriculum for Education of Disabilities in the process of specialized training for medical personnel and others		Article 6, Act on Right to Health for Persons with Disabilities
		Training and improvement of awareness in specialists in PWDs (special curriculum)	Mandatory education in treating PWDs in the process of specialized training for medical personnel and others		Article 6, Act on Right to Health for Persons with Disabilities
		Training and improvement of awareness in specialists in PWDs (medical personnel)	Obligation to offer programs on disability education for medical personnel when certifying medical institutions		Article 6, Act on Right to Health for Persons with Disabilities
Practice of healthy lifestyle	Non-smoking	Current smoking rate	Rate of PWDs aged 12 and older who currently smoke cigarettes daily or occasionally	Health Plan 2020	
		Smoking cessation attempts by current smokers	Proportion of smokers aged 12 and older who have quit smoking for more than one day in the last year to those who quit smoking	Health Plan 2020	

Area	Sub-area	Indicator	Definition of indicator	Source to construct the indicators	
				Existing policy indicators	Newly developed performance indicators
	Moderation in drink	Annual high-risk drinking rate of current drinkers	Rate of PWDs aged 12 and older who have had alcohol during the last year and had over 7 glasses per occasion for men (5 for women), or who drink more than twice a week	Health Plan 2020	
		A month's drinking rate	Percent of people over 12 years of age who have been drinking more than once a month in the past year	Health Plan 2020	
	Physical activity	Physical activity practice rate	Rate of PWDs who practice physical exercise (those who exercise more than two to three times a week for 30 minutes or more per week for activities other than rehabilitation)	Health Plan 2020 and 4th Comprehensive Plan for Persons with Disabilities	
	Nutrition	Regular meal rate	Percentage of PWDs who eat at a fixed time	Health Plan 2020	
	Accidents/poisoning	Prevalence of accidents/poisoning	Incidence of accidents or poisoning that had to be treated at hospitals or emergency rooms for the past one year after disability onset		Risk factor faced by PWDs in Korea
	Health Checkup	Rate of health checkup of PWDs	Whether a health checkup has been conducted during the last two years	Health Plan 2020	
		Rate of cancer screening of PWDs	Whether a cancer screening has been conducted during the last two years		Risk factor faced by PWDs in Korea
**Management of chronic diseases**	Obesity	Prevalence of obesity	Prevalence of obesity in PWDs aged 20 and older	Health Plan 2020	
	Hypertension	Prevalence of hypertension	Percentage of PWDs diagnosed with hypertension		Risk factor faced by PWDs in Korea
	Diabetes	Prevalence of diabetes	Percentage of PWDs diagnosed with diabetes		Risk factor faced by PWDs in Korea
	Oral health	Prevalence of dental caries	Decayed-Missing-Filled-Teeth index		Risk factor faced by PWDs in Korea
		Prevalence of periodontal disease	Community Periodontal Index of Treatment Needs		Risk factor faced by PWDs in Korea
		Level of oral hygiene	Patient Hygiene Performance index		Risk factor faced by PWDs in Korea
		Level of oral care	Number of brushing times during the day		Risk factor faced by PWDs in Korea
	Mental health	Depression level	Whether one experienced sadness or despair enough to interfere with daily life for more than two consecutive weeks for the past one year		Risk factor faced by PWDs in Korea
		Stress level	Level of stress experienced in daily life		Risk factor faced by PWDs in Korea
		Level of suicide attempts	Whether one has attempted suicide in the last year		Risk factor faced by PWDs in Korea

Area	Sub-area	Indicator	Definition of indicator	Source to construct the indicators	
				**Existing policy indicators**	**Newly developed performance indicators**
**Quality of life**	Life satisfaction	Level of satisfaction in life	Percentage of PWDs who are "very satisfied" or "satisfied" with life	Health Plan 2020	
**Children with disabilities**	Pediatric development	Implementation of tracking inspection support	Support for follow-up inspection of infants who are diagnosed with "tracking inspection required" during infant examination every two to three months		Article 6, Act on Right to Health for Persons with Disabilities
		Financial support for early detection of developmental disabilities	Financial support for the early detection of developmental disabilities in infants and young children who were asked to receive "in-depth examination"		Article 6, Act on Right to Health for Persons with Disabilities
	Diagnosis of autism spectrum disorder in early childhood	Diagnosis of autism spectrum disorder in early childhood (average age)	Average age of diagnosis of the neuropsychiatric code in Korean standard disease sign classification		Article 6, Act on Right to Health for Persons with Disabilities
		Diagnosis of autism spectrum disorder in early childhood (awareness)	Enhancing public awareness of the importance of early diagnosis and improved awareness of autism spectrum disorder in children		Article 6, Act on Right to Health for Persons with Disabilities
**Women with disabilities**	Health	Regular screening rate during pregnancy	Percentage of women with disabilities who received at least one regular screening after pregnancy was confirmed		Article 6, Act on Right to Health for Persons with Disabilities
		Infant mortality rate	Number of deaths (within one year after birth) divided by number of births in the year shown per 1,000 births		Article 6, Act on Right to Health for Persons with Disabilities
		Maternal mortality rate	Number of maternal deaths per 100,000 births		Article 6, Act on Right to Health for Persons with Disabilities
	Cancer screening	Rate of breast cancer screening	Whether a breast cancer screening has been conducted on women with disabilities over the age of 40 years during the last two years		Article 6, Act on Right to Health for Persons with Disabilities
		Rate of cervical cancer screening	Whether a cervical cancer screening has been conducted on women with disabilities over the age of 40 years during the last two years		Article 6, Act on Right to Health for Persons with Disabilities
	Sex education	Sexual education experience	Percentage of women with disabilities who have received sex education (pregnancy, giving birth, birth-control, etc.)		Article 6, Act on Right to Health for Persons with Disabilities

*Health Plan 2020: The 4th Comprehensive Plan for National Health Promotion in Korea.

*Act on Right to Health for Persons with Disabilities: Act on Guarantee of Right to Health and Access to Medical Service for Persons with Disabilities.
