# Supplementary material for: Developing key performance indicators for guaranteeing right to health and access to medical service for persons with disabilities in Korea: Using a modified Delphi
Source: PLoS One. 2018 Dec 7;13(12):e0208651. doi: 10.1371/journal.pone.0208651 (PMC6286001; doi:10.1371/journal.pone.0208651)
Supplement: S2 Table — (PDF) [file pone.0208651.s002.pdf]

S2 Table. First, second, and third Delphi survey results.

Area	Sub-area	Indicator	Definition of indicator	1st Delphi survey results			2nd Delphi survey results					3rd Delphi survey results		
				CVI		Survey results	CVI		Survey results	Modified content		CVI		Survey results
				Importance	Possibility		Importance	Possibility		Indicator	Definition of indicator	Importance	Possibility	
Health care management	Basis for improving the level of health of PWDs	Establishment of statistics on the health of PWDs (nationally approved statistics)	Expansion of health statistics for PWDs among nationally approved statistics	1.00	0.72		1.00	0.62				1.00	0.83	adopted
		Establishment of statistics on the health of PWDs (statistics on characteristics of disabilities)	Establishment of health statistics reflecting types of disability	1.00	0.76		1.00	0.66				1.00	0.83	adopted
	Medical accessibility for PWDs	Proportion of public health centers	Percentage of public health centers that conduct community-based rehabilitation projects among public health centers nationwide	0.62	0.86		0.72	1.00				0.83	1.00	adopted
		Beneficiary service rate for PWDs in residents	Percentage of PWDs receiving community-based rehabilitation services from residences	0.76	0.72		0.90	0.93	adopted					
		Establishment of facilities for PWDs in health care facilities (buildings)	Information desk for PWDs and common checklists	0.79	0.93		0.86	0.93	adopted					
		Establishment of facilities for PWDs in health care facilities (personnel)	Satisfaction of PWDs' accompanying services using volunteer workforce of health care institutions	0.79	0.79		0.83	0.90	adopted					
	Accessibility to health-related information for PWDs	Strengthening provision of health information services	Development and provision of standardized health information contents for PWDs	0.69	0.62		0.93	0.93	adopted					
	Training and education specialists	Training and improvement of awareness of specialists in PWDs (regular curriculum)	Curriculum for understanding and education of disabilities in the process of specialized training for medical personnel and others	0.86	0.86	adopted								

Area	Sub-area	Indicator	Definition of indicator	1st Delphi survey results			2nd Delphi survey results					3rd Delphi survey results		
				CVI		Survey results	CVI		Survey results	Modified content		CVI		Survey results
				Importance	Possibility		Importance	Possibility		Indicator	Definition of indicator	Importance	Possibility	
		Training and improvement of awareness in specialists in PWDs (special curriculum)	Mandatory education in treating PWDs in the process of specialized training for medical personnel and others	0.86	0.86	adopted								
		Training and improvement of awareness in specialists in PWDs (medical personnel)	Obligation to offer programs on disability education for medical personnel when certifying medical institutions	0.93	0.93	adopted								
**Practice of healthy lifestyle**	Non-smoking	Current smoking rate	Rate of PWDs aged 12 and older who currently smoke cigarettes daily or occasionally	0.72	0.66		0.76	0.79				0.83	0.90	adopted
		Smoking cessation attempts by current smokers	Proportion of smokers aged 12 and older who have quit smoking for more than one day in the last year to those who quit smoking	0.55	0.55		0.72	0.79				0.72	0.83	dropped
	Moderation in drink	Annual high-risk drinking rate of current drinkers	Rate of PWDs aged 12 and older who have had alcohol during the last year and had over 7 glasses per occasion for men (5 for women), or who drink more than twice a week	0.62	0.59		0.69	0.72				0.83	0.83	adopted
		A month's drinking rate	Percent of people over 12 years of age who have been drinking more than once a month in the past year	0.55	0.59		0.66	0.79				0.59	0.83	dropped
	Physical activity	Physical activity practice rate	Rate of PWDs who practice physical exercise (those who exercise more than two to three times a week for 30 minutes or more per week for activities other than rehabilitation)	0.97	0.62		0.97	0.69				0.93	0.93	adopted
	Nutrition	Regular meal rate	Percentage of PWDs who eat at a fixed time	0.66	0.55		0.69	0.72	modified	Number of meals per day	Number of meals per day for PWDs	0.83	0.86	adopted

Area	Sub-area	Indicator	Definition of indicator	1st Delphi survey results			2nd Delphi survey results					3rd Delphi survey results		
				CVI		Survey results	CVI		Survey results	Modified content		CVI		Survey results
				Importance	Possibility		Importance	Possibility		Indicator	Definition of indicator	Importance	Possibility	
	Accidents/ poisoning	Prevalence of accidents/poisoning	Incidence of accidents or poisoning that had to be treated at hospitals or emergency rooms for the past one year after disability onset	0.79	0.66		0.90	0.79				0.83	0.90	adopted
	Health Checkup	Rate of health checkup of PWDs	Whether a health checkup has been conducted during the last two years	0.93	0.90	adopted								
		Rate of cancer screening of PWDs	Whether a cancer screening has been conducted during the last two years	0.90	0.90	adopted								
**Management of chronic diseases**	Obesity	Prevalence of obesity	Prevalence of obesity in PWDs aged 20 and older	0.90	0.79		1.00	0.83	adopted					
	Hypertension	Prevalence of hypertension	Percentage of PWDs diagnosed with hypertension	0.86	0.79		1.00	0.90	adopted					
	Diabetes	Prevalence of diabetes	Percentage of PWDs diagnosed with diabetes	0.86	0.76		1.00	0.90	adopted					
	Oral health	Prevalence of dental caries	Decayed-Missing-Filled-Teeth index	0.86	0.68		0.93	0.75				0.83	0.90	adopted
		Prevalence of periodontal disease	Community Periodontal Index of Treatment Needs	0.86	0.54		0.93	0.71				0.83	0.90	adopted
		Level of oral hygiene	Patient Hygiene Performance index	0.75	0.54		0.86	0.79				0.83	0.90	adopted
		Level of oral care	Number of brushing times during the day	0.71	0.43	dropped								
		Rate of dental examinations	Whether an oral check-up has been conducted during the last 2 years			added	0.89	1.00	adopted					
		Sealant utilization rate	Sealant utilization rate (6-18 years old)			added	0.57	0.75	dropped					
		Scaling utilization rate	Scaling utilization rate (over 20 years old)			added	0.61	0.89	dropped					
	Mental health	Depression level	Whether one experienced sadness or despair enough to interfere with daily life for more than two consecutive weeks for the past one year	0.93	0.55		0.93	0.55				0.97	0.55	adopted[†]

Area	Sub-area	Indicator	Definition of indicator	1st Delphi survey results: CVI Importance	1st Delphi survey results: CVI Possibility	1st Delphi survey results: Survey results	2nd Delphi survey results: CVI Importance	2nd Delphi survey results: CVI Possibility	2nd Delphi survey results: Survey results	2nd Delphi survey results: Modified content Indicator	2nd Delphi survey results: Modified content Definition of indicator	3rd Delphi survey results: CVI Importance	3rd Delphi survey results: CVI Possibility	3rd Delphi survey results: Survey results
		Stress level	Level of stress experienced in daily life	0.86	0.55		0.97	0.55				0.90	0.52	adopted†
		Level of suicide attempts	Whether one has attempted suicide in the last year	0.90	0.59		0.97	0.59				0.97	0.62	adopted†
**Quality of life**	Life satisfaction	Level of satisfaction in life	Percentage of PWDs who are “very satisfied” or “satisfied” with life	0.86	0.66		0.90	0.66				0.90	0.83	adopted
**Children with disabilities**	Pediatric development	Implementation of tracking inspection support	Support for follow-up inspection of infants who are diagnosed with “tracking inspection required” during infant examination every two to three months	0.90	0.76		0.93	0.69	modified	Rate of follow-up examinations	The rate of follow-up examinations conducted on infant and toddlers who were asked to return for a “follow-up”	0.97	0.83	adopted
		Financial support for early detection of developmental disabilities	Financial support for the early detection of developmental disabilities in infants and young children who were asked to receive “in-depth examination”	1.00	0.83	adopted								
	Diagnosis of autism spectrum disorder in early childhood	Diagnosis of autism spectrum disorder in early childhood (average age)	Average age of diagnosis of the neuropsychiatric code in Korean standard disease sign classification	0.66	0.66		0.83	0.69				0.90	0.69	adopted†
		Diagnosis of autism spectrum disorder in early childhood (awareness)	Enhancing public awareness of the importance of early diagnosis and improved awareness of autism spectrum disorder in children	0.76	0.62		0.86	0.72				0.93	0.72	adopted†
	Medical examination	Rate of medical examination	Whether a medical examination has been conducted on students with disabilities attending specialized schools						added			1.00	1.00	adopted

Area	Sub-area	Indicator	Definition of indicator	1st Delphi survey results			2nd Delphi survey results					3rd Delphi survey results		
				CVI		Survey results	CVI		Survey results	Modified content		CVI		Survey results
				Importance	Possibility		Importance	Possibility		Indicator	Definition of indicator	Importance	Possibility	
Women with disabilities	Health	Regular screening rate during pregnancy	Percentage of women with disabilities who received at least one regular screening after pregnancy was confirmed	0.90	0.83	adopted								
		Infant mortality rate	Number of deaths (within one year after birth) divided by number of births in the year shown per 1,000 births	0.76	0.86		0.93	0.83	adopted					
		Maternal mortality rate	Number of maternal deaths per 100,000 births	0.72	0.86		0.86	0.83	adopted					
		Prevalence of osteoporosis	Prevalence of osteoporosis in women with disabilities over 40 years of age						added			0.86	0.83	adopted
	Cancer screening	Rate of breast cancer screening	Whether a breast cancer screening has been conducted on women with disabilities over the age of 40 during the last two years	0.90	0.86	adopted								
		Rate of cervical cancer screening	Whether a cervical cancer screening has been conducted on women with disabilities over the age of 40 during the last two years	0.90	0.86	adopted								
	Sex education	Sexual education experience	Percentage of women with disabilities who have received sex education (pregnancy, giving birth, birth-control, etc.)	0.97	0.62		1.00	0.59				0.97	0.59	adopted[†]

[*]Indicators were adopted only if the CVI value was 0.8 or greater in both importance and possibility of the indicator. $CVI \geq 0.8$ (adopted), $0.5 \leq CVI < 0.8$ (mediated differences of opinions), $CVI < 0.5$ (dropped)

[†]Although CVI value for possibility was somewhat low, it was adopted as a final performance indicator in agreement with the opinion that it was an important indicator.
